# The role of infiltrating lymphocytes in the neo-adjuvant treatment of women with HER2-positive breast cancer

**DOI:** 10.1007/s10549-021-06244-1

**Published:** 2021-05-13

**Authors:** A. J. Eustace, S. F. Madden, J. Fay, D. M. Collins, E. W. Kay, K. M. Sheehan, S. Furney, B. Moran, A. Fagan, P. G. Morris, A. Teiserskiene, A. D. Hill, L. Grogan, J. M. Walshe, O. Breathnach, C. Power, D. Duke, K. Egan, W. M. Gallagher, N. O’Donovan, J. Crown, S. Toomey, B. T. Hennessy

**Affiliations:** 1 National Institute for Cellular Biotechnology, Dublin City University, Dublin, Ireland; 2 Data Science Centre, Royal College of Surgeons in Ireland, Dublin, Ireland; 3 Department of Histopathology, Royal College of Surgeons in Ireland, Dublin, Ireland; 4 Department of Surgery, Royal College of Surgeons in Ireland, Dublin, Ireland; 5 Department of Physiology, Royal College of Surgeons in Ireland, Dublin, Ireland; 6 Conway Institute, University College Dublin, Dublin, Ireland; 7 Department of Medical Oncology, Beaumont Hospital, Dublin, Ireland; 8 Cancer Trials Ireland, Dublin, Ireland; 9 Department of Medical Oncology, St Vincent’s University Hospital, Dublin, Ireland; 10 Department of Radiology, Beaumont Hospital, Dublin, Ireland; 11 Cancer Clinical Trials and Research Unit, Beaumont Hospital, Dublin, Ireland; 12 Medical Oncology Group, Department of Molecular Medicine, Royal College of Surgeons in Ireland, Dublin, Ireland

**Keywords:** HER2-positive breast cancer, Tumour infiltrating lymphocytes, T-cells, Neo-adjuvant treatment

## Abstract

**Background:**

Pre-treatment tumour-associated lymphocytes (TILs) and stromal lymphocytes (SLs) are independent predictive markers of future pathological complete response (pCR) in HER2-positive breast cancer. Whilst studies have correlated baseline lymphocyte levels with subsequent pCR, few have studied the impact of neoadjuvant therapy on the immune environment.

**Methods:**

We performed TIL analysis and T-cell analysis by IHC on the pretreatment and ‘On-treatment’ samples from patients recruited on the Phase-II TCHL (NCT01485926) clinical trial. Data were analysed using the Wilcoxon signed-rank test and the Spearman rank correlation.

**Results:**

In our sample cohort (*n* = 66), patients who achieved a pCR at surgery, post-chemotherapy, had significantly higher counts of TILs (*p* = 0.05) but not SLs (*p* = 0.08) in their pre-treatment tumour samples. Patients who achieved a subsequent pCR after completing neo-adjuvant chemotherapy had significantly higher SLs (*p* = 9.09 × 10^–3^) but not TILs (*p* = 0.1) in their ‘On-treatment’ tumour biopsies. In a small cohort of samples (*n* = 16), infiltrating lymphocyte counts increased after 1 cycle of neo-adjuvant chemotherapy only in those tumours of patients who did not achieve a subsequent pCR. Finally, reduced CD3 + (*p* = 0.04, rho = 0.60) and CD4 + (*p* = 0.01, rho = 0.72) T-cell counts in 'On-treatment' biopsies were associated with decreased residual tumour content post-1 cycle of treatment; the latter being significantly associated with increased likelihood of subsequent pCR (*p* < 0.01).

**Conclusions:**

The immune system may be ‘primed’ prior to neoadjuvant treatment in those patients who subsequently achieve a pCR. In those patients who achieve a pCR, their immune response may return to baseline after only 1 cycle of treatment. However, in those who did not achieve a pCR, neo-adjuvant treatment may stimulate lymphocyte influx into the tumour.

**Supplementary Information:**

The online version contains supplementary material available at 10.1007/s10549-021-06244-1.

## Introduction

HER2-positive breast cancer accounts for approximately 20% of all breast cancers and prior to the clinical development of trastuzumab, had the worst outcome of any breast cancer subtype [1]. However, the development of trastuzumab and the subsequent clinical trials which have tested newer HER2-targeted therapies (including lapatinib and pertuzumab) in combination with trastuzumab, have significantly improved the outcomes of women with early-stage HER2-positive breast cancer [1].

Trastuzumab, a humanized monoclonal antibody, is known to have both cytotoxic and immunological effects on tumour cells [1, 2]. In the last decade studies have identified that the localized immune environment plays an important role in determining the outcome of women with non-metastatic HER2-positive breast cancer [3, 4]. In fact studies have shown pretreatment tumour infiltrating lymphocytes (TILs) [5] and more recently stromal lymphocytes (SLs) [4] have been shown to be independent predictive markers of future pathological complete response (pCR). Whilst many studies have correlated baseline lymphocyte levels with the likelihood of subsequent pCR, very few have studied the impact of HER2-targeted therapy on the immune environment of the tumour itself. In the TCHL clinical trial (NCT01485926), which assessed TCH (docetaxel, carboplatin, and trastuzumab) and TCHL (TCH and lapatinib) in stage II-III HER-2-positive breast cancer patients, we obtained core biopsy samples from the primary tumour from consenting patients at pretreatment and at 20-days post-cycle 1 of trastuzumab-based treatment.

Using these tumour samples, we conducted TIL analysis and assessed the impact of a single dose of TCH/L chemotherapy treatment on the numbers of infiltrating lymphocytes in breast tumours. For the first time, our study identifies that immune contexture is significantly modulated in breast tumours after only 1 cycle of TCH/L chemotherapy, and this may provide clues as to how and why some patients achieve a subsequent pathological complete response (pCR).

## Materials and methods

### Patient population and samples

**Fig. 1:**
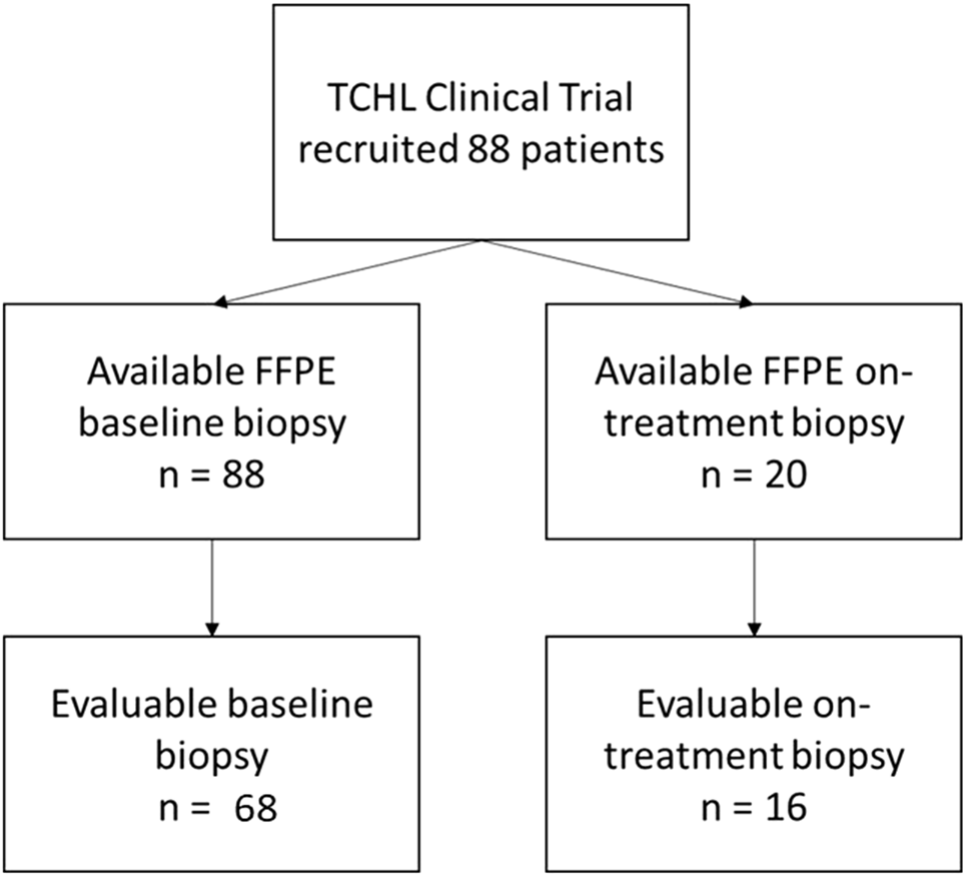
CONSORT diagram identifying which patient samples were included in the experimental analysis of the TILS in the TCHL trial (ICORG10-05) (NCT01485926) of HER2-postive breast cancer patients

TCHL (ICORG10-05) (NCT01485926) is a Phase-II neo-adjuvant study run by Cancer Trials Ireland (formerly All Ireland Co-Operative Oncology Research Group (ICORG)) assessing TCH (docetaxel, carboplatin, and trastuzumab) and TCHL (TCH and lapatinib) in stage II-III HER-2-positive breast cancer patients [6]. Full details of the trial are available at www.clinicaltrials.gov. pCR was determined in the TCHL clinical trial by the absence of invasive carcinoma. Of the 88 patients enrolled we were able to obtain lymphocyte information for 68 patients. Of those 68 patients, 20 had a core biopsy taken by an interventional radiologist, 20-days post-cycle 1 of either TCH/TCHL therapy (On-treatment samples). Samples were snap frozen and stored at −80 °C until required. Full clinicopathological details of patients involved in this study are including in Table 1, and Fig. 1 represents a consort diagram of samples used in the analysis.

**Table 1:** Full clinicopathological data of the patients recruited to the TCHL clinical samples who had samples included in the TIL analysis

	Patients with pre-treatment TIL counts (*n* = 68)		Patients with on-treatment TIL counts (*n* = 16)	
Characteristic	No. of patients	%	No. of patients	%
ER status				
Negative	28	41.2	9	56.3
Positive	40	58.8	7	43.7
PR status				
Negative	39	57.3	9	56.3
Positive	29	42.7	7	43.7
pCR				
Yes	32	47.1	9	56.3
No	36	52.9	7	43.7
Targeted therapy				
Trastuzumab	30	44.1	7	43.7
Lapatinib	10	14.7	2	12.5
Tras+Lap	28	41.2	7	43.7
Age, years				
< 49	35	51.5	10	62.5
≥ 49	33	48.5	6	37.5
Tumour size, cm				
≤ 5	46	67.6	11	68.8
> 5	17	25	4	25
Unknown	5	7.4	1	6.2
N stage				
N0	20	29.4	3	18.8
N1	40	58.8	11	68.8
N2	1	1.5	1	6.2
NX	2	2.9	1	6.2
Unknown	5	7.4	0	0
M stage				
M0	68	100	16	100
Overall stage				
IIA	26	38.2	7	43.8
IIB	25	36.8	6	37.5
IIIA	4	5.9	1	6.2
IIIB	8	11.8	0	0
IIIC	0	0	0	0
Unknown	5	7.3	2	12.5

### Sample processing

Baseline tumour biopsies obtained prior to neo-adjuvant chemotherapy were formalin fixed and paraffin embedded (FFPE). Haematoxylin and Eosin (H&E) staining was performed on 3 µM sections of biopsies and assessed for invasive tumour epithelial cellularity by a Histopathologist. Only samples with greater than 10% tumour cellularity were used for further analysis. On-treatment samples were embedded in optical coherence tomography and the samples were cryo-sectioned. A single 3 µM section was taken for H&E staining and analysis, and the adjacent ten 10 µm sections were cut and stored in a chilled cryovial. Following this, a second 3 µM section was then cut for H&E staining. Cut sections were stored at −80 °C.

### Immunohistochemistry (IHC) and TIL counting

H&E staining was performed on a Thermo Shandon Varistain Gemini stainer using Harris haematoxylin (CellPath, RBA-4213-00A) and alcoholic eosin Y (Thermo Scientific, 6766008) before being cover slipped (Thermo Shandon Consul). 4 µm serial tissue sections were cut using a Leica RM2135 microtome. IHC analysis was carried out on a Bond-III immunostainer (Leica Biosystems, Newcastle, UK). Primary antibodies CD45, Common Leukocyte Antigen (Dako, Clones 2B11 + PD7/26, M0701) and Cytokeratin (Dako, Clone AE1/3, M3515) were diluted in Bond Primary Antibody Diluent (Leica, AR9352) at 1/500 and 1/400, respectively. Pre-treatment of samples was carried out on the Bond-III using Bond Epitope Retrieval Solution I (Leica, AR9961) for 30 min (CD45) and Bond Enzyme Pre-treatment solution (Leica, AR9551) for 10 min (AE1/3). Detection and visualisation of stained cells was achieved using the Bond Polymer Refine Detection Kit (Leica, DS9800) with Bond DAB Enhancer (Leica, AR9432). Tissues were counterstained with haematoxylin and cover slipped. Slides were scanned at 40X using a Philips 2.0 scanner, viewed with Philips Image Management System 2.2 and analysed as per current guidelines [7]. As per the recommendations of the TIL working group [7, 8] which stated that TILs at the invasive edge or intra-tumoural TILs can still be included for research purposes, we proceeded with a research study to assess the impact of TCHL treatment on TILs in HER2-positive breast cancer. To that end, four random areas the size of 1 high power microscope field (between 100,000 and 100,500uM^2^) were selected in each case. CD45 + cells were counted in each of the four areas. Cytokeratin AE1/3 was used to assess the location of tumour cells relative to the CD45 + cells in each of the areas counted. These IHC stains were completed on FFPE baseline biopsy samples (*n* = 68/88) and on fresh frozen (FF) biopsies taken 20-days post-cycle 1 (Day-20) of TCH/TCHL (*n* = 20/88). A lymphocyte was counted as a TIL if it was observed to be in direct contact with an invasive tumour epithelial cell [7]. A stromal lymphocyte (SL) was determined if it was dispersed in the stroma, with no contact between the tumour epithelium and the lymphocyte [7]. Overall Lymphocyte count (OL) was the combined TIL and SL count. TIL analysis was independent of treatment groups. In samples where the tumour had completely regressed following treatment, the number of lymphocytes were assessed by counting four random high power fields. In the instances of no residual tumor in on-treatment biopsy samples, it is important to note that the biopsy samples were small. Whilst we report no residual tumor it may be that any residual tumor was so scattered and minimal, that it was not captured in the small biopsy.

### T-cell IHC and image analysis

We had previously shown from MCP counter analysis [9] a small subset of TCHL patient samples that increased levels of T-cells were associated with response to TCHL-based therapy [10]. We had sufficient material from 13 patients who had matched pre and on-treatment biopsies to perform T-Cell IHC and image analysis. 3 µm serial tissue sections were cut using a Leica RM2135 microtome. IHC analysis was carried out on a Bond-III immunostainer (Leica Biosystems, Newcastle, UK). Primary antibodies for CD3 (Leica, NCL-L-CD3-565), CD4 (Leica, NCL-CD4-368) and CD8 (Leica, NCL-CD8-4B11) were diluted in Bond Primary Antibody Diluent (Leica, AR9352) at 1/40, 1/100 and 1/100, respectively. Pre-treatment of samples was carried out on the Bond-III using Bond Epitope Retrieval Solution I (Leica, AR9961) for 20 min (CD3, CD8) and using Bond Epitope Retrieval Solution II (Leica, AR9640) for 20 min (CD4). Detection and visualization of stained cells was achieved using the Bond Polymer Refine Detection Kit (Leica, DS9800) with Bond DAB Enhancer (Leica, AR9432). Tissues were counterstained with haematoxylin and cover slipped. The CD3, CD4 and CD8 stained slides for 13 cases (pre-treatment and on-treatment) were scanned at 40X using a Philips 2.0 scanner and the whole section analysed using the open access image analysis software QuPath [11]. The positive cell detection tool was used to measure the number of positive cells per square millimeter of tissue and compared against the assessment of a Histopathologist. Two comparisons were made using both QuPath and the Histopathologist: Firstly, for each antibody, the number of positive cells in the pre-treatment biopsy was compared to the post-treatment biopsy and secondly the number of CD4 + and CD8 + cells were compared between the pre-treatment biopsy and the post-treatment biopsy. Due to the large number of positive cells in most samples the pathologist score could not be given as a numerical value but was noted as a comparative statement between the samples being analyzed. The QuPath results for all samples were then compared to the pathologist score to ensure accuracy of the software, and QuPath results were then used for quantitative analysis.

### Statistical analysis

The non-parametric Wilcoxon signed-rank test was used to determine if there was a significant difference between pathological complete response (pCR) and no-pCR for the three comparison groups (TILs; SLs and overall lymphocytes). The test was paired when comparing baseline and on-treatment groups. The paired test was also used when comparing pre versus On-Treatment CD3 + , CD4 + and CD8 + counts. T-cell markers and tumour content were correlated using the non-parametric Spearman’s rank correlation. Tumour content versus T-cell markers was plotted and loess regression was used to fit a smooth line to illustrate the relationship between the two variables. P-values of less than 0.05 were considered statistically significant.

## Results

### Pre-treatment TIL levels correlate with a better pCR rate

**Fig. 2:**
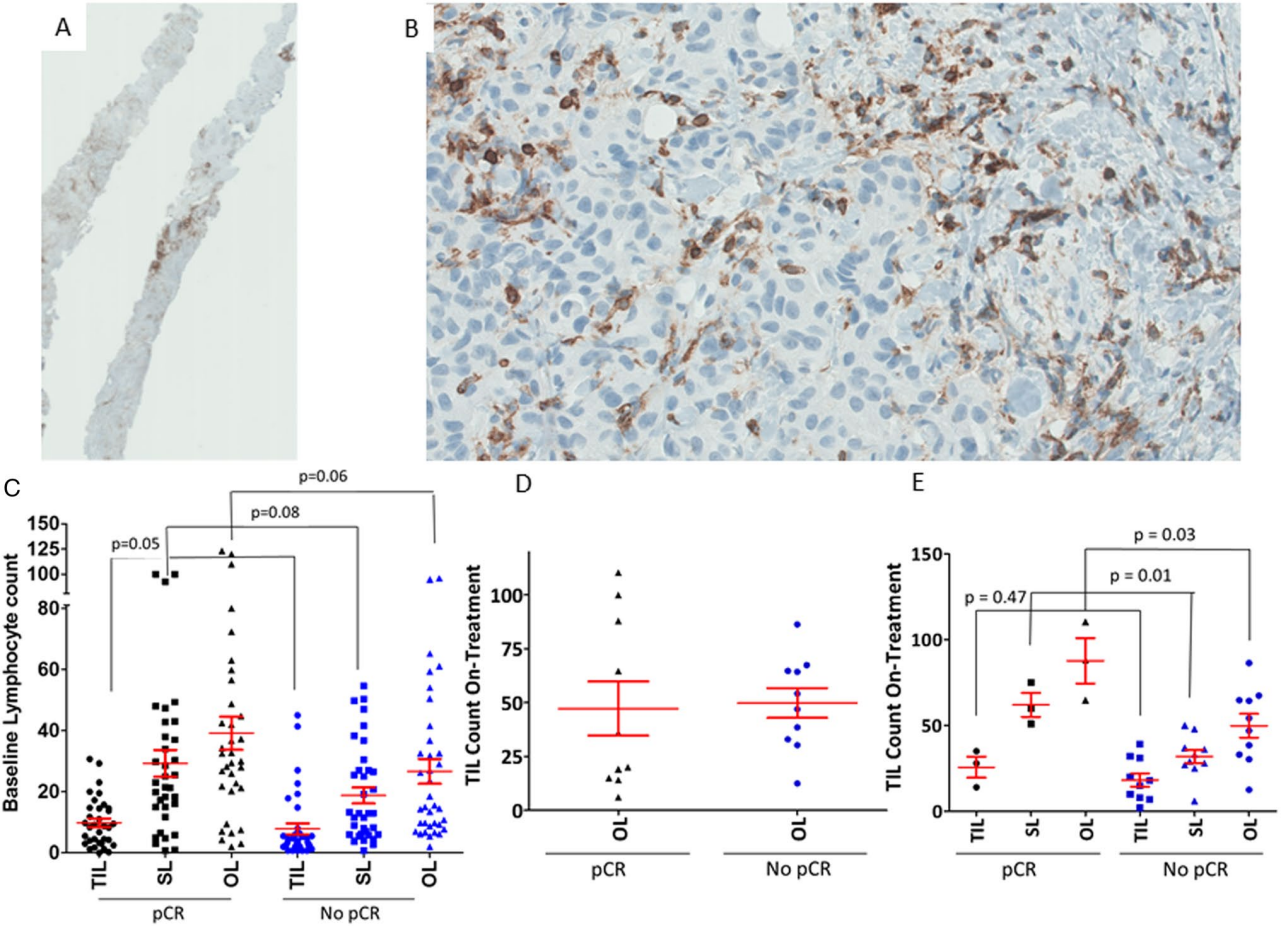
Analysis of TIL content by AE1/AE3 and CD45 staining in TCHL neo-adjuvant treated clinical trial baseline pre-treatment biopsy samples from HER2-positive breast cancer patients **a** 2X overview of CD45 staining in a FFPE baseline biopsy sample **b** 40X magnification of CD45 staining showing lymphocyte content. (Four random areas the size of a high power microscope field (between 100,000 and 100,500μM^2^) were selected in each case for TIL analysis.) **c** Correlation between baseline counts of TILs, SLs and OLs with pCR status in TCHL trial patients (*n* = 68/88). **d** Correlation between pCR status and lymphocyte counts in On-treatment biopsy samples obtained at 20-days or after 1 cycle of neo-adjuvant treatment (*n* = 20). **e** Correlation between pCR status and lymphocyte counts in those On-treatment biopsy samples where residual tumour remains after 20-days of neo-adjuvant treatment (*n* = 13). p-values are calculated using a Wilcoxon signed-rank test and a p-value < 0.05 was considered significant. PCR-Pathological complete response; *No pCR* no pathological complete response, *TIL* tumour infiltrating lymphocyte, *SL* stromal lymphocytes, *OL* overall lymphocytes

We determined the number of both SLs and TILs in the baseline pretreatment FFPE tumors of 68/88 patients who were recruited to the TCH/L trial (Fig. 2a, b). Our study demonstrated that patients who achieved a pCR at surgery post-chemotherapy had significantly higher numbers of TILs (*p* = 0.05) in their baseline pre-treatment tumour samples, relative to those patients who did not achieve a pCR post-chemotherapy (Fig. 2c). We also observed that pre-treatment SL counts may be predictive of a better chance of achieving a pCR post-chemotherapy but did not reach statistical significance (*p* = 0.08). While larger studies have shown estrogen receptor status is predictive of pCR, it did not have an impact on rates of pCR in the TCHL study (*p *= 0.2141) [12].

### Correlation between on-treatment lymphocyte counts and pCR

We have previously shown that tumour epithelial cells are undetectable in the day-20 On-treatment biopsies of some patients who go on to achieve pCR at subsequent surgery [13]. Tumour biopsy samples were obtained from 20 patients 20-days after they had undergone cycle 1 of neo-adjuvant chemotherapy treatment (On-treatment samples). Analysis of both SLs and TILs in these On-treatment tumour biopsy samples identified that, in contrast to the pre-treatment tumour biopsies, SL, TIL and OL counts are not significantly different between the two groups defined by pCR versus no-pCR at subsequent surgery (Fig. 1d).

In our pCR group we observed that after 1 cycle of therapy 70% (7/10) of biopsies had no residual tumour remaining (< 5% residual tumour). When we compared TIL counts in the pCR group we observed a non-significant trend whereby TIL numbers were lower in the biopsies with no residual tumour relative to the remaining biopsies where residual tumour remained (*p* = 0.14). Of the 20 available on-treatment biopsy samples, eight (which includes a sample from the no-pCR group) had no residual tumour left in the biopsy after 20-days of starting neo-adjuvant treatment. Upon excluding these cases in which the immune response is possibly already subsiding, we observed that OL (*p* = 9.09 × 10^–3^) counts were significantly higher in on-treatment tumour biopsies from patients who subsequently achieved a pCR relative to those who failed to achieve a pCR at subsequent surgery (Fig. 2e). When we stratified the lymphocyte counts into either TILs or SLs, patients who achieved a subsequent pCR after completing neo-adjuvant chemotherapy treatment had significantly higher SL counts (*p* = 9.09 × 10^–3^) in their On-treatment tumour biopsy samples than those patients who did not achieve a subsequent pCR, but this effect was not seen for TILs (*p* = 0.1).

### Level of lymphocytes increase with neo-adjuvant TCHL chemotherapy treatment

**Fig. 3:**
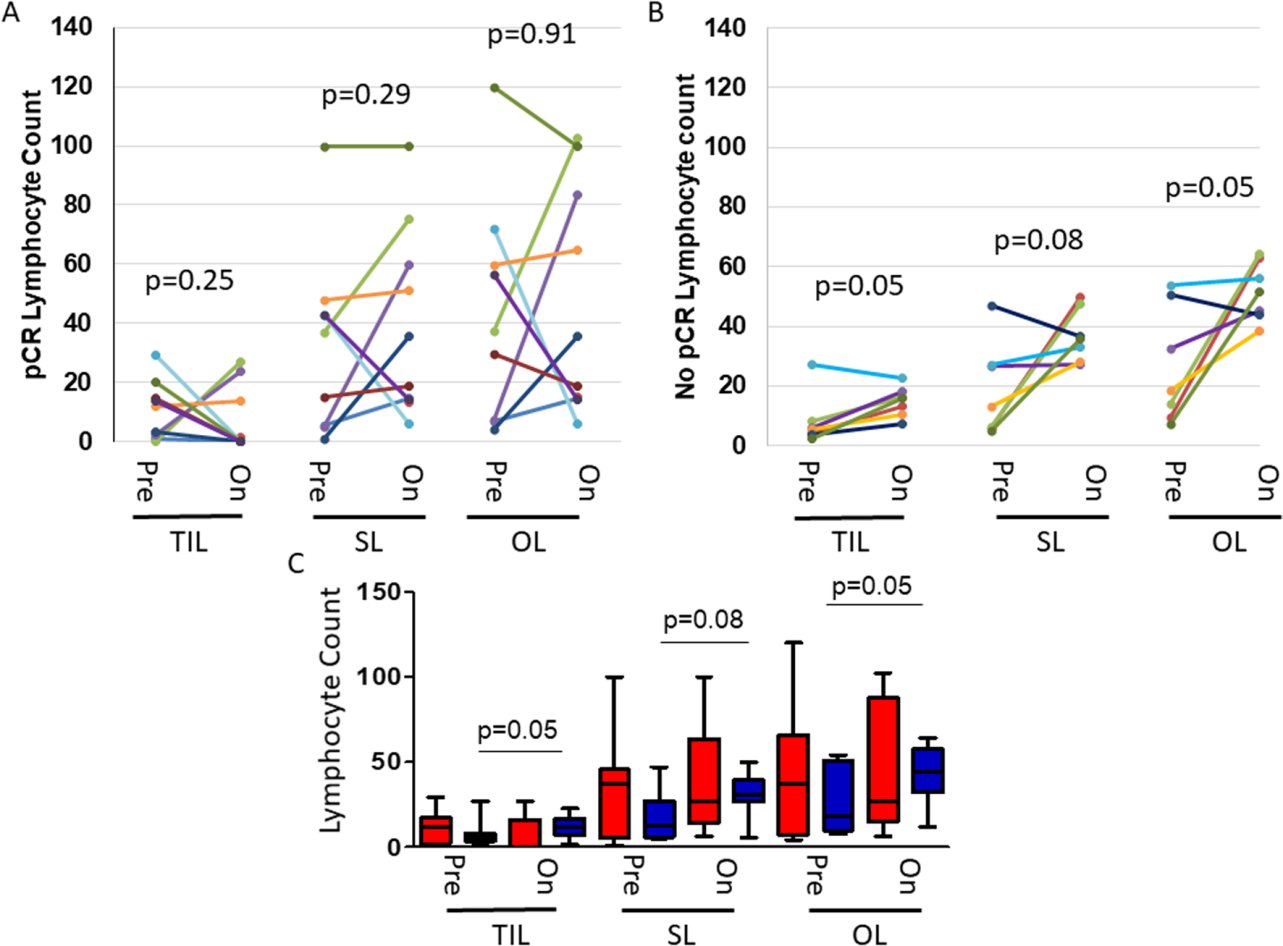
Comparison of the changes in lymphocyte counts in individual patients in baseline pre-treated biopsy samples and those with matched biopsy samples after 20 days of chemotherapy in patients who **a** those who achieved a pCR (*n* = 9) versus **b** those failed to achieve a pCR (*n* = 7). **c** Average lymphocyte counts observed between patients who achieved a pCR and those who failed to achieve a pCR. p-values are calculated using a paired Wilcoxon signed-rank test and a p-value < 0.05 was considered significant. pCR-Pathological complete response; *No pCR* no pathological complete response; *Pre* baseline biopsy; *On* on-treatment biopsy; *TIL* tumour infiltrating lymphocyte; *SL* stromal lymphocytes; *OL* overall lymphocytes; Red bars – pCR; Blue bars – no pCR

Of the 20 fresh frozen On-treatment patient samples available, 16 had matched baseline infiltrating lymphocyte information allowing for an analysis of changes in infiltrating lymphocyte levels in paired pre- and on-treatment samples. Examination of these 16 samples (pCR *n* = 9 vs No-pCR *n* = 7), irrespective of residual tumour status, determined that 1 cycle of neo-adjuvant TCHL treatment was associated with changes in levels of infiltrating lymphocytes in patient tumours when they were stratified on the basis of subsequent pCR. There was no consistent significant difference in TIL, SL or OL levels between baseline and day-20 tumour biopsies when the tumours in the group that attained a pCR at subsequent surgery were analysed (Fig. 3a). However, in patients who did not achieve a subsequent pCR, we observed a trend from the matched baseline to the day-20 On-treatment samples whereby lymphocyte numbers in the tumours significantly increased (TILs, *p* = 0.05; SILs, *p* = 0.08; OLs, *p* = 0.05) (Fig. 3b, c).

### Neo-adjuvant TCHL treatment reduces number of tumour-related T-cells

Given the key role of T-cells in regulating the immune response, we performed IHC analysis on 13/16 paired pre- and on-treatment fresh frozen samples for which we had sufficient material. The T-cell markers CD3 (pan T-cell), CD4 (T helper cells) and CD8 (cytotoxic T-cell) were examined. No distinction was made between stromal and tumour infiltrating T-cell populations. When analysing all patients, we observed that levels of CD3 + , CD4 + or CD8 + T-cells did not significantly increase or change from the baseline to the On-treatment tumour biopsy samples (Supplementary Figure 1).

**Fig. 4:**
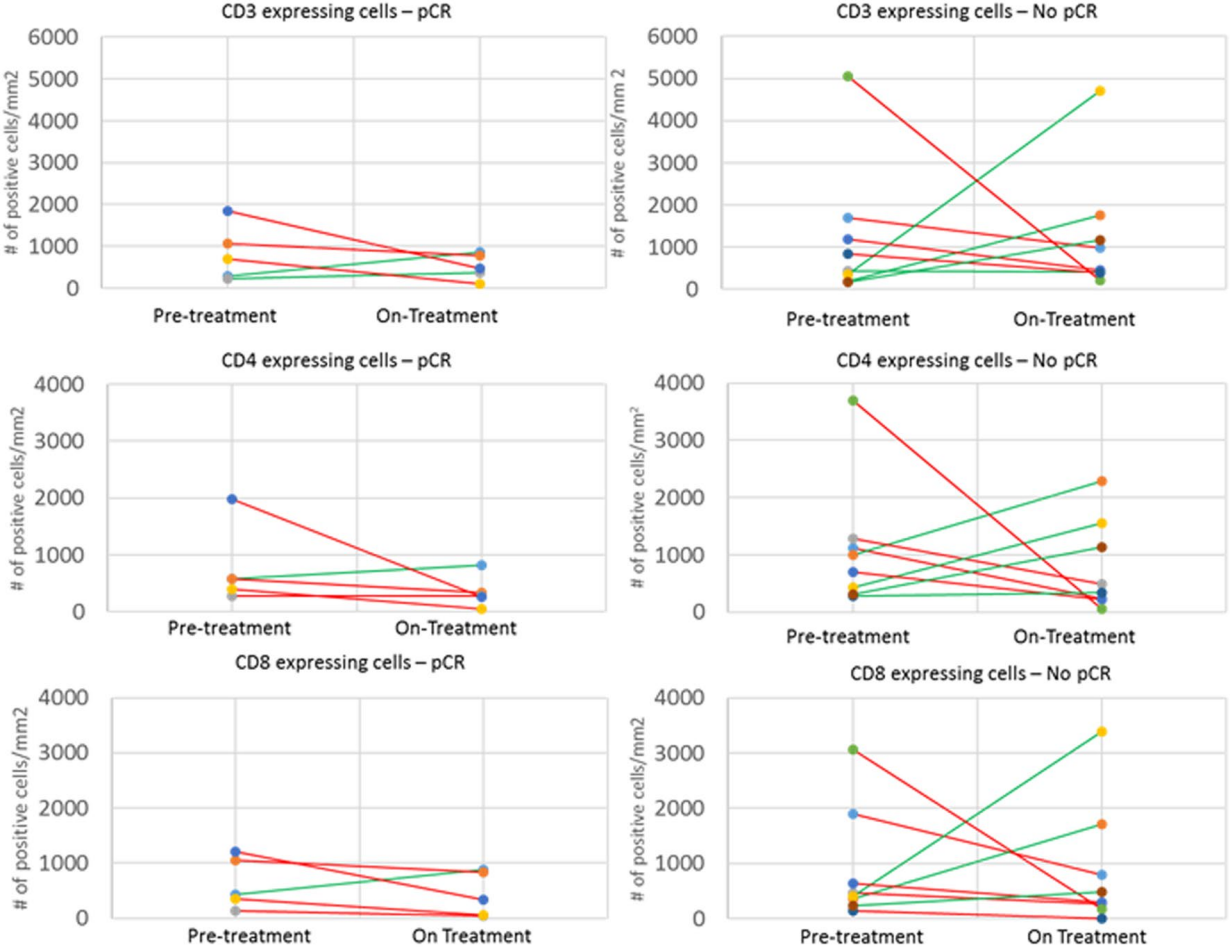
Comparative expression of CD3+ , CD4+ and CD8+ stained cells in breast cancer tumour samples taken from pre-treated and post-20-days of TCHL treatment. The CD3 + , CD4 + and CD8 + stained slides for pre-treatment and post-treatment were scanned using a Philips 2.0 scanner and were analyzed using the open access image analysis software QuPath. ‘Red’ lines indicate a decrease in levels of CD3 + , 4 + , 8 + T-cells ,respectively, between pre and post-treatment samples, whilst ‘green’ lines indicate an increase in CD3 + , 4 + , 8 + T -cell numbers

To further analyse the effect of neo-adjuvant treatment on T-cell numbers we observed the changes in CD3 + , CD4 + and CD8 + T-cells in the matched baseline and day-20 On-treatment tumour biopsy samples of individual patients (Fig. 4). Interestingly, in those patients who achieved a subsequent pCR, we found a decrease in levels of CD4 + or CD8 + T-cells in 4/5 patients at day-20. However, in those patients who did not achieve a subsequent pCR, only 4/8 patients had a decrease in CD4 + T-cells at day-20, whilst 5/8 had a decrease in CD8 + T-cells.

### *A reduction in tumour volume correlates with decreased numbers of CD4* + *and CD3* + *T-cells*

**Fig. 5:**
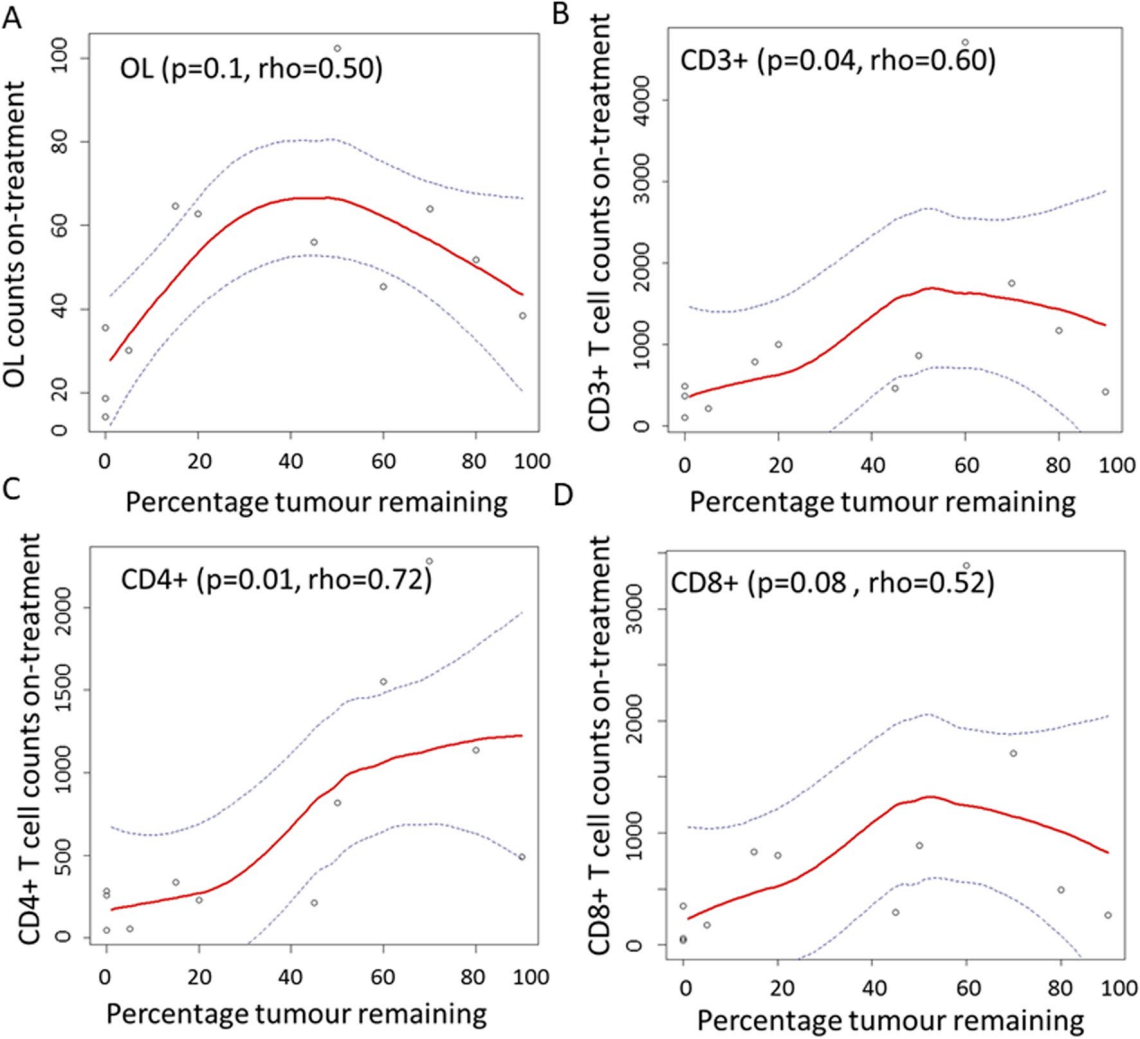
T-cell count versus the residual tumour post-1 cycle of TCH/L treatment. **a** OL **b** CD3+  **c** CD4+  and **d** CD8+T-cells. The Spearman Rank Correlation rho and accompanying p-values are shown on the plots. Loess regression was used to fit the smooth line to the data (red) and the dotted lines show the 95% confidence intervals

As outlined above, neo-adjuvant TCH/L-based treatment results in a reduction of tumour volume after 1 cycle of treatment, and this tumour reduction correlates with a greater chance of a patient achieving a pCR [13]. However, we aimed to further understand if there was a correlation between loss of lymphocytes, either CD3+ , CD4 + or CD8 + T-cells in the day-20 On-treatment tumour biopsy and a reduction in tumour volume in the biopsy (Fig. 5). Using a Spearman rank correlation, we found a significant correlation whereby a reduction of CD3 + (*p* = 0.04, rho = 0.60) and CD4 + (*p* = 0.01, rho = 0.72) T-cells was associated with decreased residual tumour content post-1 cycle of treatment. We also observed a similar positive trend for CD8 + T-cells, but the results did not reach statistical significance (*p* = 0.08, rho = 0.52). We, however, did not see a positive trend for OLs (*p* = 0.1, rho = 0.50).

## Discussion

Long-term outcomes for women with HER2–driven early-stage breast cancer have significantly improved since the advent of HER2-targeted therapies including trastuzumab and lapatinib [1]. In our study, we wanted to determine the impact, not just of TILs on likelihood of pCR, but also of neo-adjuvant TCH/L treatment on the levels of TILs in patient samples. Therefore, uniquely, as part of the TCHL trial we were able to obtain on-treatment biopsy samples taken after 20-days of neo-adjuvant chemotherapy. Using these on-treatment samples we aimed to define the impact of neo-adjuvant treatment not only on infiltrating lymphocyte counts but also specifically on T-cell numbers. The goal being to determine if changes in lymphocyte populations might influence a patient’s chance of achieving a subsequent pCR.

TILs are proven to have positive prognostic implications in the outcome of early-stage breast cancer [14]; with elevated levels of TILs associated with a greater chance of a patient achieving a pCR (*n* = 1256) [3]. This effect occurs regardless of the type of neo-adjuvant anti-HER2 agent or chemotherapy used [3]. In our study, we aimed to determine for research purposes the impact of neoadjuvant treatment on TILs in HER2-positive breast cancer as per the TIL working group [7] and Vinayak et al. [8]. In the TCHL study, we found that baseline numbers of OLs in the tumour were not a significant predictive indicator of pCR (although there was a clear trend (*p* = 0.0634)). Importantly, we classified these lymphocytes in accordance with Salagado et al*.* [7], being dependent on their proximity to the tumour and then defined them as either TILs (in contact with tumour) or SLs (not in contact with tumour epithelial cells). We determined that, at diagnosis, it is a higher number of TILs that most determine the likelihood of achieving a pCR. The lack of correlation between SLs and likelihood of pCR in our study is in contrast to that observed in other HER2-positive or triple negative breast cancer studies, where SLs are an independent predictive marker of pCR [15, 16]. The discrepancy between these results could be reflective of the relatively small sample size in all studies, and would have to be examined in a larger cohort.

In a limited number of the day-20 on-treatment biopsies available, we determined SL and TIL levels in patients who achieved a pCR (but had residual tumour after 1 cycle of therapy) and those who failed to achieve a pCR at subsequent surgery. We determined that SL numbers were significantly increased at day-20 in the pCR group. However, we also found, by comparing matched lymphocyte levels between baseline and on-treatment tumour biopsy samples, that levels of lymphocytes do not increase in the group that achieve a pCR at subsequent surgery. This was in contrast to the non-pCR group, where both TIL and OL counts were significantly increased in the tumour after 1 cycle of neo-adjuvant chemotherapy treatment. Our findings support the hypothesis that in patients who achieve a pCR the immune microenvironment which already surrounds the tumour at baseline likely plays an important role in response to subsequent therapy. Hamy et al*.* [17] identified that increased numbers of SLs at surgery are associated with a worse outcome. Our results are supported by the TRIO-US B07 study (NCT00769470), which included a similar treatment schedule to our TCHL clinical trial [18]. Hurvitz et al., demonstrated that on-treatment stromal TIL numbers were higher (but not significantly *p* = 0.066) in the pCR group relative to the non-pCR group [18]. Therefore the results of both the TRIO-US B07 study and our TCHL study identifies that treatment may quickly increase the numbers of lymphocytes around a tumour, in particular in patients who do not achieve a subsequent pCR, and thus also suggests that analysis of lymphocyte numbers after the start of a patient’s treatment may provide a good indication as to how a patient’s tumour is likely to respond to treatment.

Interestingly, in the only other neo-adjuvant HER2-positive breast cancer study to collect matched on-treatment biopsy samples (PAMELA), Nuciforo et al*.* [19] found that in patients with HER2-positive breast cancer who achieved a pCR, 15 days of treatment with dual HER2-blockade (but no chemotherapy) resulted in a significant increase in the level of TILs, and this effect was associated with an increased chance of achieving a subsequent pCR. However, in the PAMELA study, in contrast to our study, they did not determine SLs or TILs as separate populations, but only counted OLs. In our clinical study, patients were also treated with chemotherapy along with trastuzumab ± lapatinib, which may have a different impact on the immune contexture within tumours.

Many studies have identified the importance of baseline lymphocyte numbers as a positive prognostic factor in determining subsequent pCR [20–22]. To date, no study has looked at the impact of neo-adjuvant treatment on immune contexture within tumours, particularly T-cell levels, and how this correlates with future pCR. The pan T-cell marker CD3 indicates the T-cell numbers present in the biopsy samples as opposed to the CD45 IHC antibody which identifies a broad array of hematopoietic immune cell types including T-cells, NK-cells, B-cells and macrophages/monocytes (but not erythrocytes and platelets) [23]. CD4 and CD8 identify two important general T-cell subsets. CD4 + T-cells can play an important role in directly killing tumour cells, influencing the active immune response within the tumour microenvironment, and increasing the activity of B-cells and cytotoxic CD8 + T-cells in secondary lymphoid organs [24]. CD8 + T-cells are antigen-specific, cytotoxic cells that are a major effector cell of the adaptive immune response [25]. Exhausted CD8 + T-cells are the target of immune checkpoint inhibitor drugs such as pembrolizumab, nivolumab, and ipilimumab that are producing remarkable responses across multiple cancer types [26]. When we compared matched baseline and on-treatment samples in our small cohort of samples, we observed a reduction in numbers of CD4 + and CD8 + T-cells, in particular in tumours in the pCR group. Indeed, overall we found a significant correlation between a reduction in CD3 + and CD4 + T-cell numbers and a reduction in tumour cell content in tumour biopsies after 1 cycle of treatment. The latter is correlated with likelihood of subsequent pCR at surgery, as we have also shown previously [13]. Reduced TILs or in our case reduced CD4 + T-cell numbers around the tumour could be a direct result of chemotherapy treatment [17, 27–30], but the association with reduced tumour cell content here suggests an already diminishing immune response in those tumours that are exquisitely sensitive to neo-adjuvant treatment. That the change in CD8 + T-cells is not as dramatic may reflect a different clearance dynamic between the T-cell subsets following tumour elimination. Supportive of this result, the TRIO-US B-07 study showed that levels of CD8 + T cells were lower in those tumours which had reduced immune content, likely as a result of reduced tumour burden [18]. However, the TRIO-US B-07 study did not correlate this result with subsequent pCR. Our results, however, are based on a small cohort of samples therefore further classification of subsets of CD4 + T-cells, such as the immune dampening CD4 + FOXP3 + regulatory T-cells (Tregs), in a larger population of on-treatment HER2 + breast cancer biopsy samples in the future is warranted to provide greater insight [31].

Our analysis sheds light on the modulation of the immune response that occurs early during neo-adjuvant chemotherapy. In on-treatment tumour biopsy samples, lymphocyte counts increase after 1 cycle of neo-adjuvant therapy (in particular in tumours that do not end in pCR at subsequent surgery), but a reduction in T-cell counts occurs in some tumours which correlates with a lower tumour burden in day-20 on-treatment tumour biopsies. The latter is associated with a higher likelihood of pCR at subsequent surgery [13]. The results of our study and the TRIO-US study indicate that even after 1 cycle of treatment, the immune system may have already ‘played its role’ in responding tumours. Our results are limited by small tumour numbers but highlight the need to study the early impact of neo-adjuvant treatment in a larger population to confirm these exciting initial findings. These studies could be expanded to assess the impact of dual anti-HER2 antibody therapy (including both trastuzumab and pertuzumab) on immune contexture.

## Supplementary Information

Below is the link to the electronic supplementary material.

Supplementary file1 (TIF 92 kb)

## Data Availability

All data will be made available under reasonable request.
