# Supplementary figures and images for: The role of infiltrating lymphocytes in the neo-adjuvant treatment of women with HER2-positive breast cancer
Source: Breast Cancer Res Treat. 2021 May 13;187(3):635–45. doi: 10.1007/s10549-021-06244-1 (PMC8197702; doi:10.1007/s10549-021-06244-1)

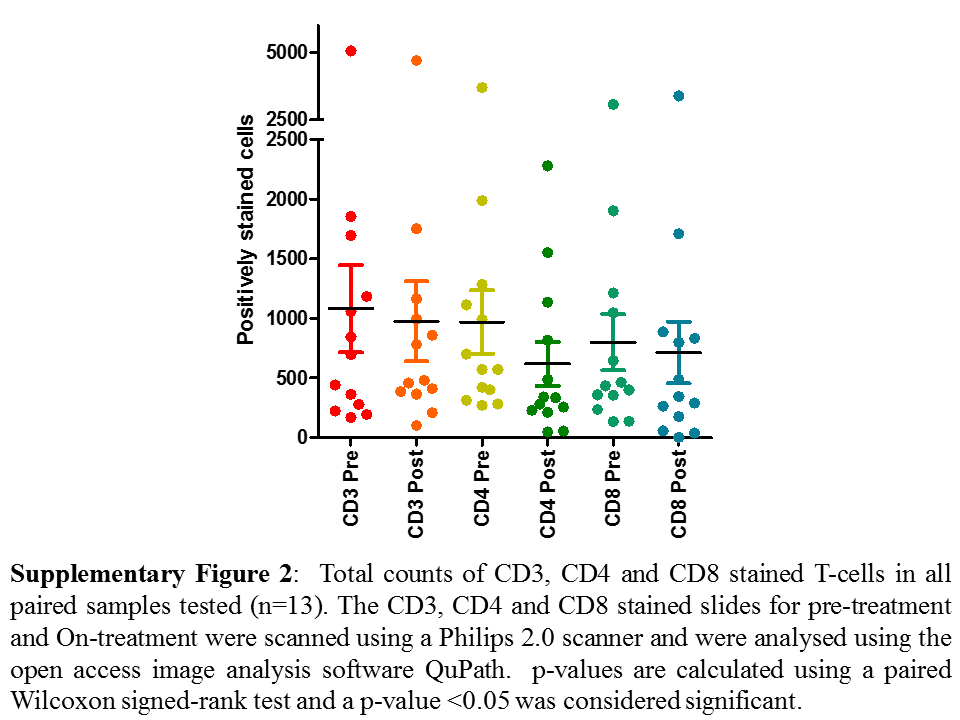

Supplement: Supplementary file 1 — Supplementary file1 (TIF 92 kb) [file 10549_2021_6244_MOESM1_ESM.tif]
